# Efficacy and safety of radiofrequency treatment for improving knee pain and function in knee osteoarthritis: a meta-analysis of randomized controlled trials

**DOI:** 10.1186/s13018-021-02906-4

**Published:** 2022-01-15

**Authors:** Jian Liu, Ting Wang, Zhen-Hua Zhu

**Affiliations:** Department of Emergency, The First Hospital of Hunan University of Chinese Medicine, No. 95 Shaoshan Middle Road, Changsha, 410007 Hunan Province China

**Keywords:** Radiofrequency ablation, Knee osteoarthritis, Meta-analysis, Pain score, Knee function

## Abstract

**Background:**

The clinical utility of radiofrequency (RF) in patients with knee osteoarthritis (OA) remains unclear. We conducted a meta-analysis to systematically evaluate the efficacy and safety of RF treatment in patients with knee OA.

**Methods:**

Searches of the PubMed, Web of Science, EMBASE, Cochrane Library, China National Knowledge Infrastructure, and Wanfang Data databases were performed through August 30, 2021. The major outcomes from published randomized controlled trials (RCTs) involving patients with knee OA were compared between RF and control groups, including Visual Analogue Scale (VAS) or Numerical Rating Scale (NRS) scores, the Western Ontario and McMaster Universities Osteoarthritis Index (WOMAC), Oxford Knee Score (OKS), Global Perceived Effect (GPE) scale, and adverse effects at available follow-up times.

**Results:**

Fifteen RCTs involving 1009 patients were included in this meta-analysis, and the results demonstrated that RF treatment correlated with improvements in pain relief (VAS/NRS score, all *P* < 0.001) and knee function (WOMAC, all *P* < 0.001) at 1–2, 4, 12, and 24 weeks after treatment as well as patients’ degree of satisfaction with treatment effectiveness (GPE scale, 12 weeks, *P* < 0.001). OKSs did not differ significantly between the two groups. Moreover, treatment with RF did not significantly increase adverse effects. Subgroup analysis of knee pain indicated that the efficacy of RF treatment targeting the genicular nerve was significantly better than intra-articular RF at 12 weeks after treatment (*P* = 0.03).

**Conclusions:**

This meta-analysis showed that RF is an efficacious and safe treatment for relieving knee pain and improving knee function in patients with knee OA.

**Supplementary Information:**

The online version contains supplementary material available at 10.1186/s13018-021-02906-4.

## Background

Knee osteoarthritis (OA), a degenerative joint disease of the knee, typically results in progressive loss of articular cartilage elasticity and erosion of the articular surface [1, 2]. Knee OA is most common in the elderly (> 70 years of age) with a prevalence as high as 40%, which will continue to increase as obesity rises and life expectancy is extended [3, 4]. Knee pain is the main clinical symptom of knee OA and causes functional limitations, fatigue, depressed mood, and loss of independence, which worsens over time and eventually leads to disability [5]. As the candidate treatment regimen for end-stage knee OA, arthroscopic surgery or total knee arthroplasty (TKA) provides satisfactory functional recovery [6]. However, persistent pain is not relieved effectively in approximately 20–53% of the patients undergoing arthroscopic surgery or TKA [7, 8]. In addition, some patients have contraindications or are not good candidates for surgery due to age or severe comorbidities.

Current treatments for knee OA concentrate on relieving pain, slowing cartilage destruction, and improving quality of life [9]. Various nonsurgical modalities, including physical therapy, weight loss, oral nonsteroidal anti-inflammatory drugs (NSAIDs), intra-articular corticosteroid or hyaluronic acid (HA) injections, and extracorporeal shockwave therapy, have been used for the treatment of knee OA [10, 11]. These noninvasive therapies may substantially relief pain but do not reverse the underlying disease process [12, 13]. Recently, radiofrequency (RF) treatments, including radiofrequency ablation (RFA), cooled radiofrequency ablation (CRF), and pulsed radiofrequency ablation (PRF), have been extensively used in patients with severe joint pain who refuse to undergo TKA and have provided convincing therapeutic benefits. RFA is thermally mediated to cause tissue injury within a relatively discrete homogeneous lesion [14]. The advantage of RFA is precise heating in a narrow rim (< 1 mm) of tissue that is in direct contact with the ablation electrode (≥ 45–50 °C) [15, 16]. Thus, RF treatment can improve joint function and relieve pain by delivering targeted thermal damage to genicular nerves that innervate painful tissue, thereby disrupting the transmission of pain signals [17, 18].

In recent years, several small and large randomized controlled trials (RCTs) of RF treatment for knee OA were conducted to evaluate the clinical efficiency of such treatments. Most of the obtained evidence suggests that RFA can be a safe and effective treatment for both knee pain reduction and knee functional improvement lasting between 3 and 12 months. The advent of CRF ablation and nonablative PRF therapy has further broadened the clinical utility of RF for chronic pain states, expanding beyond facet-joint-mediated pain to peripherally innervated targets [19]. Although knee RF has been the subject of numerous publications, high-quality RCTs remain sparse, and the clinical utility of RF remains ambiguous due to confounding factors from different studies. Therefore, we conducted a systematic review and meta-analysis of RCTs to evaluate RF-induced effects and safety in patients with knee OA.

## Methods

### Data sources and searches

The meta-analysis was performed in accordance with PRISMA (Preferred Reporting Items for Systematic review and Meta-Analysis) guidelines [20]. It was registered with PROSPERO (CRD42021292558). We searched the PubMed, Web of Science, EMBASE, Cochrane Library, China National Knowledge Infrastructure, and Wanfang Data databases until August 30, 2021. The following medical subject heading terms were used: “knee,” “osteoarthritis,” “radiofrequency,” “genicular nerve,” “intra-articular,” “randomized controlled trial,” “controlled clinical trial” and “humans.” No language or country limitations were applied to our meta-analysis. All RCTs in the search results were screened by two authors according to the inclusion criteria. If the article title and abstract description were ambiguous with respect to the inclusion criteria, the full text was downloaded and reviewed carefully.

### Inclusion criteria

Reported outcomes in published RCTs were recorded for RF and control groups of knee OA patients. Studies were eligible for inclusion if they met all of the following criteria: (i) patients were diagnosed with knee OA; (ii) patients in the experimental group received RF therapy (RFA, PRF, CRF, and other form); (iii) the clinical trial was designed with a control group; (iv) the study included the following outcome measurements: the Visual Analogue Scale (VAS) or Numerical Rating Scale (NRS), the Western Ontario and McMaster Universities Osteoarthritis Index (WOMAC), Oxford Knee Score (OKS), Global Perceived Effect (GPE) scale, and adverse effects at different time points after treatment; and (v) studies were RCTs.

### Exclusion criteria

The exclusion criteria for the meta-analysis were as follows: (i) patients underwent knee arthroplasty or arthroscopic surgery; (ii) full text was not available; (iii) provided unextractable or insufficient data; and (iv) case reports, abstracts, conference presentations, editorials, and expert opinions.

### Data extraction and quality assessment

Two experienced researchers independently assessed the quality of the included RCTs with the Cochrane Handbook for Systematic Reviews of Interventions, which includes 10 specific domains consisting of random sequence generation, allocation concealment (selection bias), blinding of participants and personnel (performance bias), blinding of outcome assessment (detection bias), incomplete outcome data/intention-to-treat analysis/loss to follow-up (attrition bias), compliance, and selective reporting (selection bias). Disagreements were resolved by a third person who served as an intermediary and made the final decision. Each included study was graded as having a high risk, low risk, or unclear risk of bias.

Two reviewers independently extracted relevant data from the original studies using a standardized data extraction form and clarified discrepancies by re-evaluation and discussion with the other authors. The following data were extracted for analysis: name of the first author, year of publication, country, study design, sample size, age, sex, Kellgren–Lawrence classification, mode of RF, ultrasound transducer parameters, primary outcomes such as VAS/NRS, OKS, WOMAC, and GPE scale at baseline and at available follow-up times, and adverse effects. The corresponding authors of RCTs were contacted to request missing data or clarification regarding unclear data.

### Statistical analysis

The statistical analysis was performed according to the recommendations from the Cochrane Collaboration. Weighted mean difference (WMD) and corresponding 95% confidence interval (CI) values for the difference in means were used to evaluate continuous data, and the risk difference with 95% CI was calculated for dichotomous data. Heterogeneity across studies was assessed by the Cochran Q test (significance level of *P* < 0.05) and the *I*^2^ statistic. For the *I*^2^ statistic, we considered *I*^2^ < 25% as low heterogeneity and *I*^2^ > 75% as high heterogeneity. Data were also analyzed with a fixed-effects model for *P* > 0.05 and *I*^*2*^ < 50% or a random-effects model for *P* < 0.05 and *I*^*2*^ ≥ 50%. Based on the differences in variables such as RF modes, location, intervention target, diagnosed nerve block (DNB), sex ratio among cases, and body mass index (BMI), subgroup analyses were performed. Sensitivity analysis was conducted to observe the impact of any single study on the pooled WMD. Review Manager Version 5.3 (The Nordic Cochrane Center, The Cochrane Collaboration, 2014, Copenhagen) software was used to analyze the pooled data. A two-tailed *P* value < 0.05 was considered statistically significant.

## Results

**Fig. 1:**
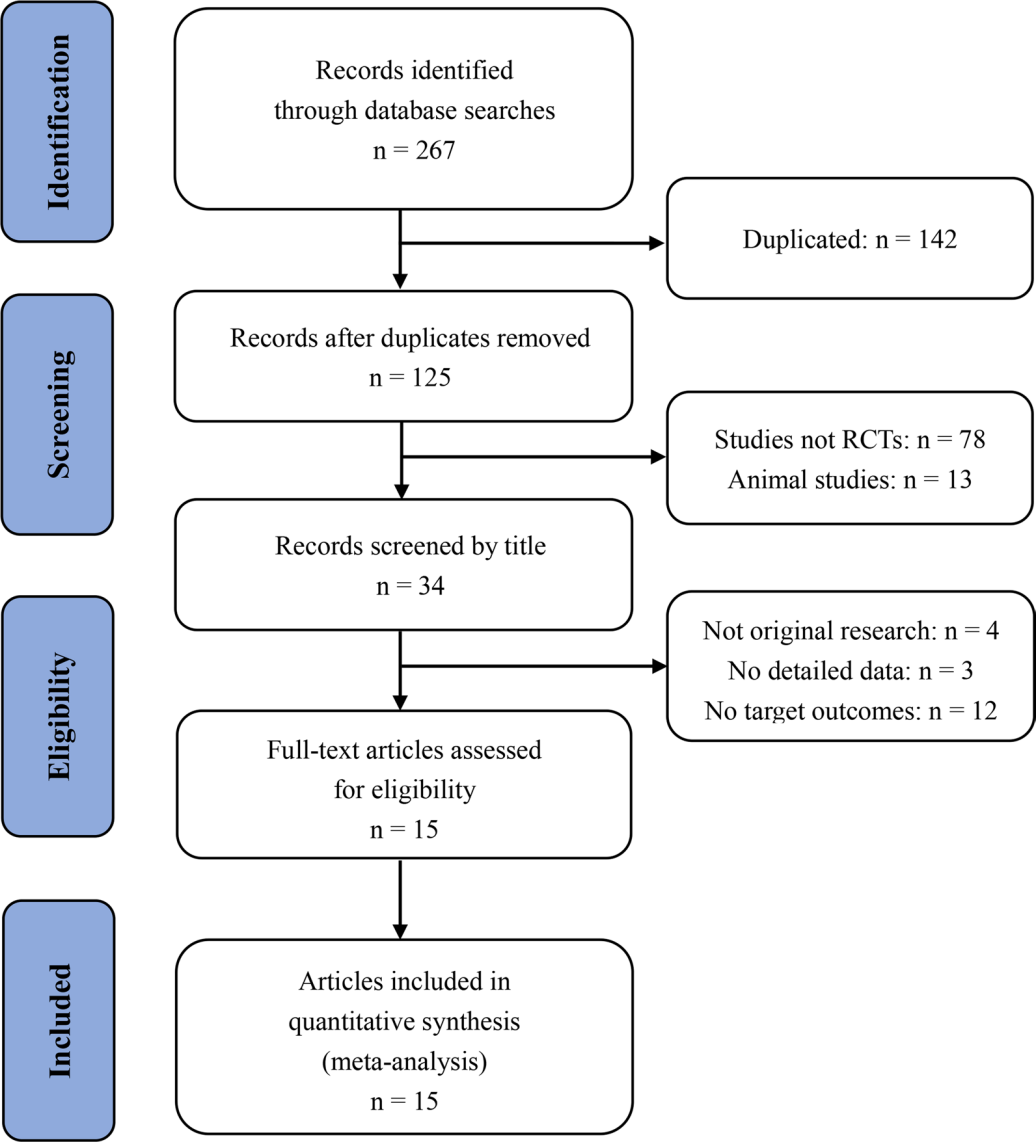
Flowchart of study selection. RCTs = randomized controlled trials

A total of 267 articles were identified by searching six electronic databases. After electronically removing 142 duplicated articles and manually excluding 91 obviously irrelevant studies upon reading the title and abstract, 34 publications were assessed in detail. From these, 19 articles were excluded for various reasons and 15 eligible trials were ultimately included for further qualitative and quantitative analysis [21–35]. The detailed screening method and results are shown in Fig. 1.

### Basic characteristics and quality assessment

The primary characteristics of the included RCTs are presented in Table 1. We included 15 studies in our meta-analysis, of which 13 were single-center studies and 2 were multi-center studies. The included RCTs were conducted in eight countries and were published between 2011 and 2021. A total of 1009 patients were enrolled from the 15 eligible trials. Of these, 503 recipients had been assigned to receive RF and were included in the RF group, and the remaining patients who were not treated with RF were assigned to the control group. The patients ranged in age from 47.8 to 70.9 years.

**Table 1:** Characteristics of each included trials

References	Country	No. of Patients (RF/Control)	Treatment Gender (M/F)	Control Gender (M/F)	Mean age (years) (RF/Control)	Kellgren-Lawrence grade	BMI (RF/Control)	Disease courses (years) (RF/Control)	Treatment pain score baseline	Control pain score baseline
[21]	Korea	17/18	2/15	3/15	67.9/66.5	NA	26.2/26.5	6.3/7.4	7.82 (1.38)	7.72 (0.75)
[22]	China	17/19	NA	NA	NA	1–3	NA	NA	7.26 (1.34)	7.19 (1.57)
[23]	Iran	24/26	11/13	10/16	57.0/60.6	3–4	NA	NA	7.08 (1.41)	7.11 (1.03)
[24]	China	31/31	NA	NA	NA	NA	NA	NA	7.25 (1.33)	7.21 (1.58)
[25]	China	27/27	7/20	9/18	62.2/62.4	2–4	NA	5.01/4.96	7.12 (1.08)	7.14 (1.03)
[35]	China	12/12	5/7	3/9	51.7/54.0	1–3	NA	NA	8.25 (0.62)	8.16 (0.72)
[26]	Turkey	37/36	7/30	9/25	64.0/64.0	2–3	23.5/22.9	5/5	NA	NA
[27]	China	45/47	17/28	20/27	66.1/65.9	NA	24.5/24.9	8.2/8.2	6.53 (1.10)	6.38 (1.03)
[28]	China	49/47	12/37	11/36	56.5/61.5	3–4	NA	3.04/2.96	7.3 (1.2)	7.3 (1.4)
[29]	Egypt	30/30	9/21	12/18	62.0/56.9	2–4	32.0/30.2	7.6/5.7	7.07 (0.2)	7.07 (0.2)
[30]	America	76/75	26/50	26/49	63.0/66.0	3–4	30.6/30.4	10.7/8.6	7.3 (1.2)	7.2 (1.0)
[31]	UK	15/15	6/9	6/9	63.0/63.0	2–4	31.0/31.0	5.6/4.3	6.3 (1.2)	5.8 (1.2)
[32]	America	89/88	37/52	34/54	63.3/63.1	2–3	32.2/30.5	7.5/8.8	NA	NA
[33]	China	26/27	10/16	12/15	59.5/60.9	3–4	24.6/25.8	2.7/2.9	6.46 (1.14)	6.37 (0.93)
[34]	Italy	8/8	2/6	3/5	70.4/70.9	NA	29.5/29.6	9.6/10.4	8.25 (0.70)	8.0 (1.19)

RF, radiofrequency; M, male; F, female; BMI, body mass index; NA, not applicable

For RF therapy, RFA was applied in 7 trials, PRF in 4 trials, CRF in 2 trials, and CRMRF and RF thermocoagulation in 1 trial, respectively. Regarding to the intervention targets, eight studies focused on the genicular nerve, and seven studies applied an intra-articular procedure. Moreover, five studies applied DNB to obtain the source of pain and position of targets for RF therapy. In addition, VAS/NRS scores were available in 12 studies for comparing the pain improvement between the two groups at different follow-up time points. The OKS and WOMAC scores were available in 4 and 5 studies, respectively, for evaluation of knee functional improvement. GPE scale data were available in 3 studies for assessing the patients’ degree of satisfaction regarding treatment effectiveness. The detailed intervention procedural parameters, results, and adverse effects are presented in Table 2.

**Table 2:** Details of intervention procedure parameters, results, adverse effects, and follow-up time of the 15 RCTs included in the current meta-analysis

References	Intervention	Control	Treatment target	Intervention parameters	Diagnostic nerve block	Scoring methods	Adverse effects	Follow-up time (weeks)
[21]	RFA	Shame-RFA	GN	70 °C, 90 s	Yes	VAS, GPE, OKS	None reported	1, 4,12
[22]	RFA	Intra-articular injection of sodium hyaluronate	GN	70 °C, 120 s	No	VAS	None reported	1, 4, 12
[23]	PRF	Intra-articular injection of dextrose	IA	42 °C, 15 min	No	VAS	None reported	1, 4,12
[24]	RFA	Intra-articular injection of sodium hyaluronate	GN	70 °C, 120 s	No	VAS, OKS	None reported	1, 4,12
[25]	RFA	Intra-articular injection of platelet-rich plasma and sodium hyaluronate	IA	70 °C, 120 s	No	VAS	NA	1, 12
[35]	PRF	Intra-articular injection of lidocaine and betamethasone	IA	42 °C, 120 s	Yes	VAS, WOMAC	None reported	1, 4, 12, 24
[26]	RFA	Intra-articular injection of bupivacaine, morphine, and betamethasone	GN	80 °C, 90 s	No	WOMAC	NA	4, 12
[27]	PRF	Oral celecoxib	IA	42 °C, 120 s	No	VAS, WOMAC	NA	4, 24
[28]	RFA	Intra-articular injection of sodium hyaluronate	GN	60, 70, and 80˚C, 90 s	No	VAS	NA	1, 12, 24
[29]	RFA	Oral paracetamol and NAIDS	GN	80 °C, 270 s	No	VAS, WOMAC	None reported	1, 12, 24
[30]	CRF	Intra-articular steroid	GN	60 °C, 150 s	Yes	NRS, OKS	34/30	4, 12, 24
[31]	CRMRF	Shame-CRMRF	IA	15 min	No	VAS	None reported	1, 4,12
[32]	CRF	Intra-articular injection of sodium hyaluronate	IA	60 °C, 150 s	Yes	GPE, WOMAC	18/9	4, 12, 24
[33]	RF thermocoagulation	Intra-articular steroid	GN	70 °C, 120 s	No	GPE	None reported	1, 4, 12, 24
[34]	PRF	Shame-PRF	IA	42 °C, 120 s	Yes	NRS, OKS	None reported	1, 4, 12, 24

RFA, radiofrequency ablation; PRF, pulsed radiofrequency ablation; CRF, cooled radiofrequency ablation; CRMRF, capacitive resistive monopolar radiofrequency; NSAIDs, nonsteroidal anti-inflammatory drugs; GN, genicular nerve; IA, intra-articular; VAS, Visual Analogue Scale; GPE, Global Perceived Effect; WOMAC, Western Ontario and McMaster Universities; OKS, Oxford Knee Scores; NA, not applicable

**Fig. 2:**
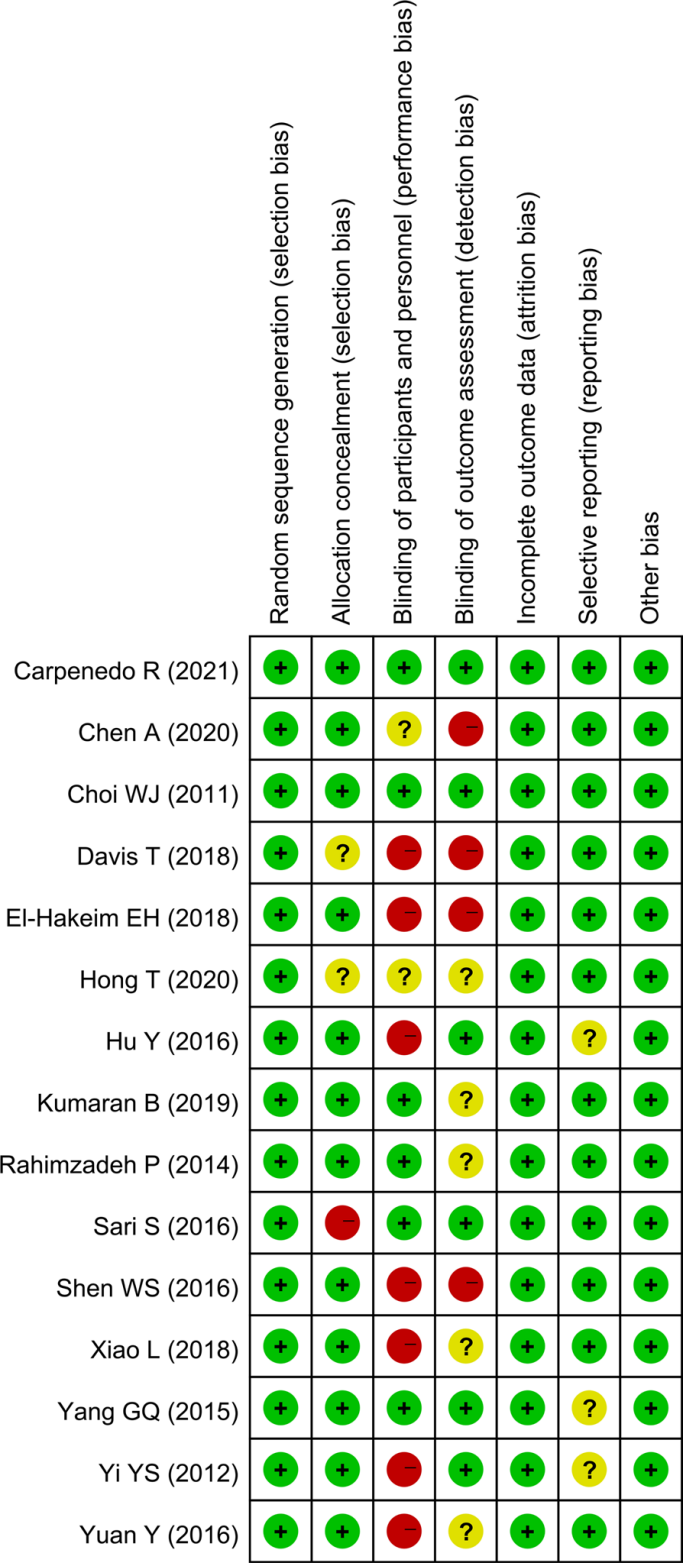
Risk of bias summary

**Fig. 3:**
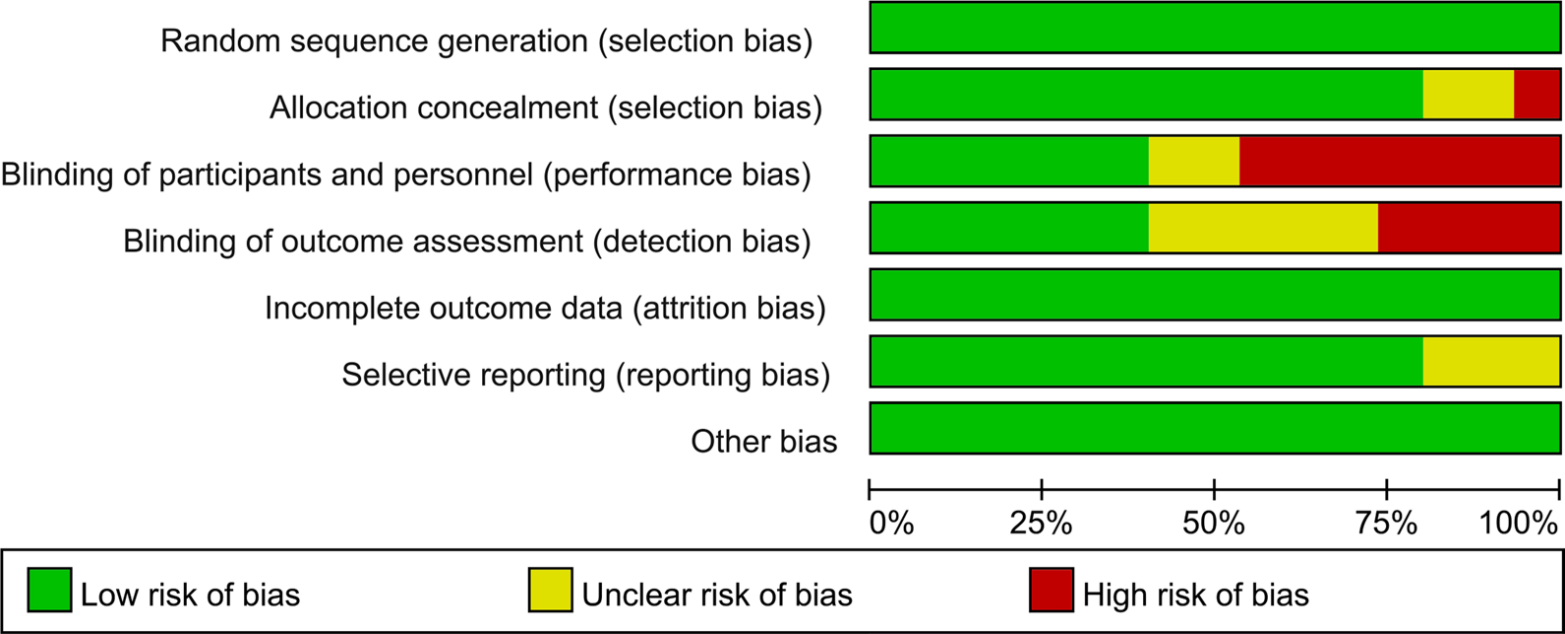
Risk of bias graph

Two authors independently assessed the quality of each RCT. Among the 15 RCTs, 12 trials had relatively high methodological quality and met allocation concealment criteria. The overall details of quality assessment are shown in Figs. 2 and 3.

### Pain score

**Fig. 4:**
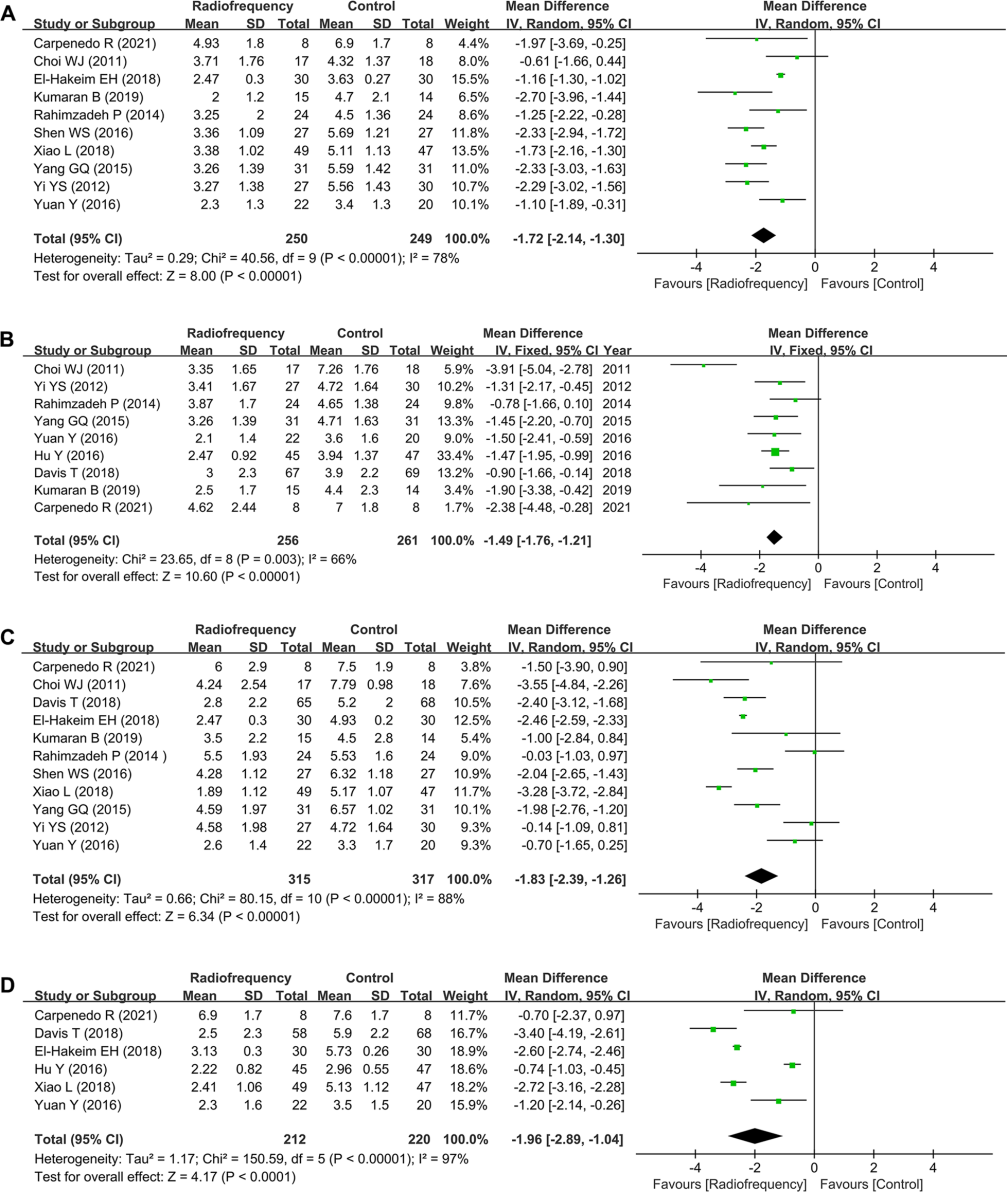
Forest plots for the assessment of pain scores between the radiofrequency group and control group. At **A** 1–2 weeks, **B** 4 weeks, **C** 12 weeks, and **D** 24 weeks. SD, standard deviation; CI, confidence interval

Twelve trials with 706 patients reported the outcome of pain score on VAS or NRS, with 10 RCTs reporting scores at 1–2 weeks, 9 RCTs reporting scores at 4 weeks, 11 RCTs reporting scores at 12 weeks, and 6 RCTs reporting scores at 24 weeks (Fig. 4). Because the pooled results from data at four time points exhibited significant heterogeneity, the random-effects model was used to obtain WMDs and the corresponding 95% CIs (all *I*^*2*^ > 50%, *P* < 0.05). Meta-analysis indicated that significant pain relief was achieved at four follow-up time points by the application of RF compared with that of patients in the control group (1–2 weeks, WMD = − 1.72, 95% CI − 3.96 to − 0.1.44, *P* < 0.001; 4 weeks, WMD = − 1.49, 95% CI − 1.76 to − 1.21, *P* < 0.001; 12 weeks, WMD = − 1.83, 95% CI − 2.39 to − 1.26, *P* < 0.001; 24 weeks, WMD = − 1.96, 95% CI − 2.89 to − 1.04, *P* < 0.001).

### OKS/WOMAC scores

**Fig. 5:**
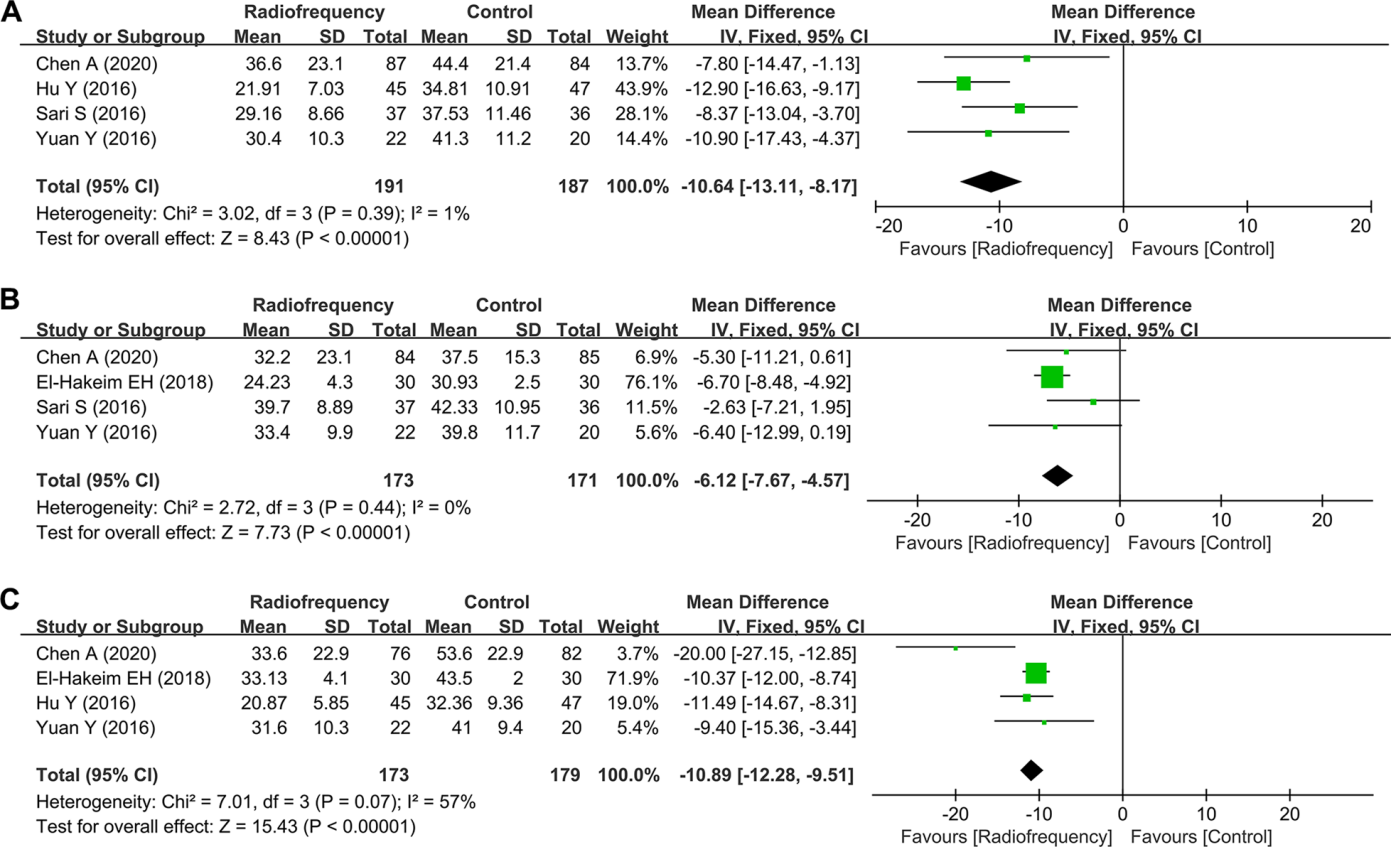
Forest plots for the assessment of WOMAC index between the radiofrequency group and control group. At **A** 4 weeks, **B** 12 weeks, and **C** 24 weeks. WOMAC, Western Ontario and McMaster Universities Arthritis Osteoarthritis Index; SD, standard deviation; CI, confidence interval

Due to significant between-study heterogeneity for OKS, the random-effects model was applied, except that the fixed-effects model was used at 1–2 weeks after treatment (Additional file 1: Fig. S1). Notably, the majority of results for OKS indicated insignificant knee function improvement with RF treatment (1–2 weeks, WMD = − 2.73, 95% CI − 5.10 to − 0.37, *P* = 0.02; 4 weeks, WMD = − 2.15, 95% CI − 9.51 to 5.21, *P* = 0.57; 12 weeks, WMD = − 0.23, 95% CI − 11.24 to 10.77, *P* = 0.97). However, the pooled results for WOMAC score at three time points exhibited no significant heterogeneity, and the fixed-effects model was used to obtain WMDs and the corresponding 95% CIs (all *I*^*2*^ < 50%, *P* > 0.05; Fig. 5). The pooled results showed that RF treatment significantly improved knee function (4 weeks, WMD = − 10.64, 95% CI − 13.11 to − 8.17, *P* < 0.001; 12 weeks, WMD = − 6.12, 95% CI − 7.67 to − 4.57, *P* < 0.001; 24 weeks, WMD = − 10.89, 95% CI − 12.28 to − 9.51, *P* < 0.001, respectively).

### GPE scale

**Fig. 6:**
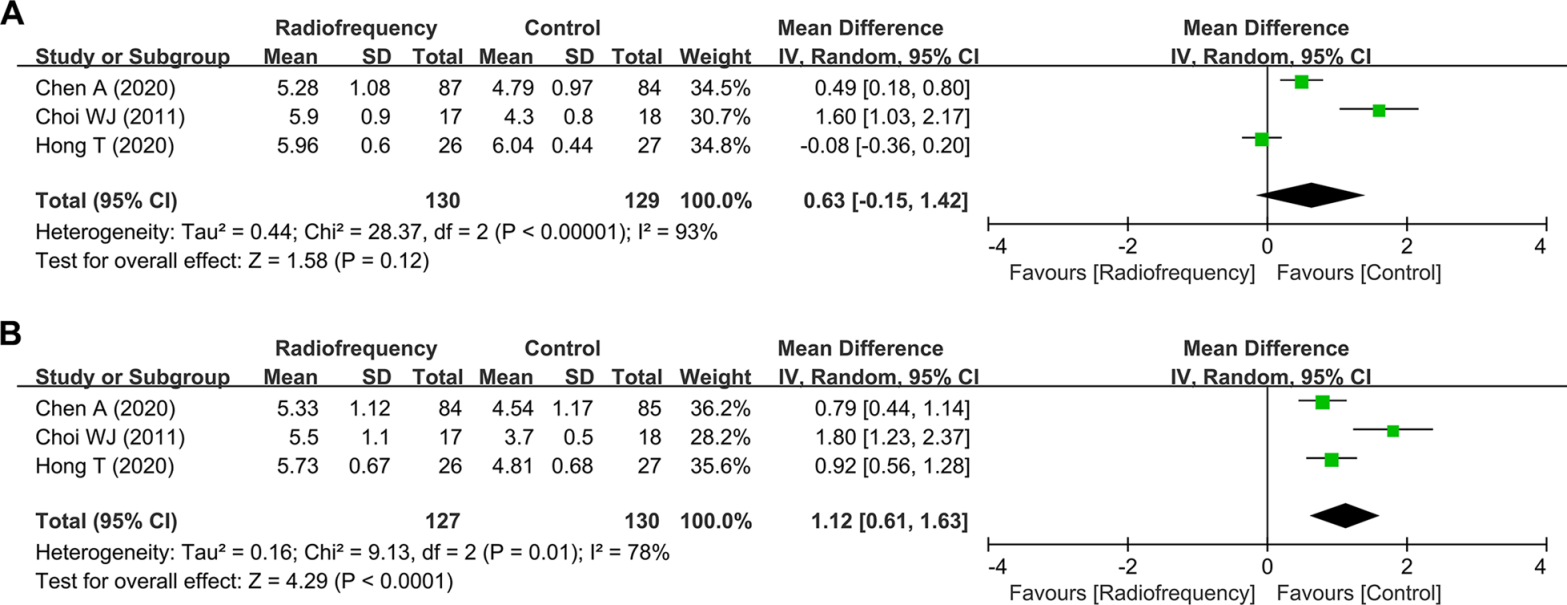
Forest plots for the assessment of GPE scale between the radiofrequency group and control group. At **A** 4 weeks and **B** 12 weeks. GPE, Global Perceived Effect; SD, standard deviation; CI, confidence interval

Three studies reported the outcome of GPE scale, which also showed significant heterogeneity (4 weeks, *I*^*2*^ = 93%, *P* < 0.001; 12 weeks, *I*^*2*^ = 78%, *P* = 0.01, respectively). Therefore, the random-effects model was used (Fig. 6). The pooled results indicated no significant difference at 4 weeks after treatment and a significant difference at 12 weeks between the two groups (4 weeks, WMD = − 0.63, 95% CI − 0.15 to 1.42, *P* = 0.12; 12 weeks, WMD = 1.12, 95% CI 0.61 to 1.63, *P* < 0.001).

### Adverse effects

**Fig. 7:**
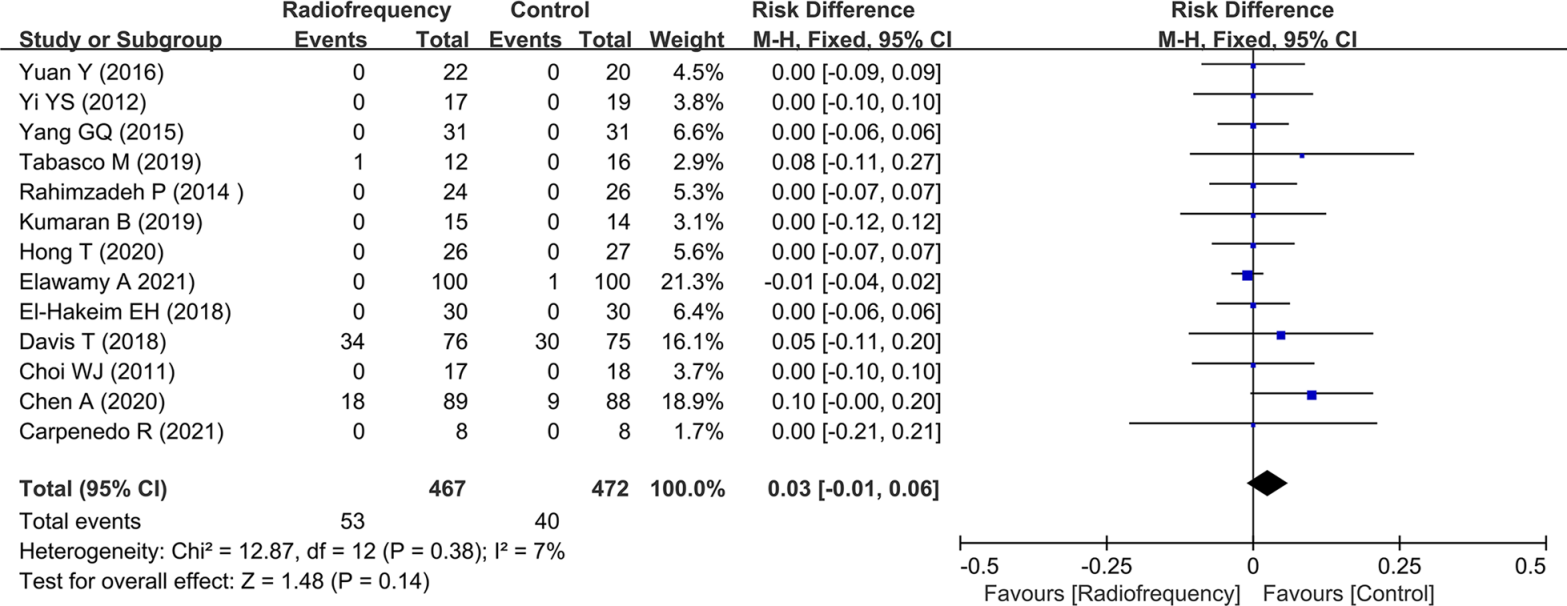
Forest plots for the assessment of adverse effects between the radiofrequency group and control group. RD, risk difference; CI, confidence interval

Adverse events induced by RF were reported in 91 patients in two RCTs and were not serious (Fig. 7). The majority of these adverse events were deemed unrelated to the study intervention. Davis et al. reported that three patients in the CRF group experienced four severe adverse events (SAEs), whereas seven patients in the control group experienced eight SAEs. However, they illustrated that none of the SAEs were related to the study treatments. In addition, no adverse events were reported in 9 studies. There was no significant heterogeneity among the 12 studies (*I *^*2*^ = 17%, *P* = 0.28), and a fixed-effects model was used. Based on the available data, the use of RF treatment did not significantly increase adverse effects (risk difference 0.03, 95% CI − 0.01 to 0.06, *P* = 0.14).

### Subgroup analysis

Subgroup analysis was conducted to find the sources of heterogeneity for pain score associated with the pooled results. The following subgroup analyses were performed: 1) with or without RFA treatment; 2) location (Asia vs. others); 3) application of DNB before treatment or not; 4) treatment target of genicular nerve or other; 5) ratio of females/males ≥ 2 or not; and 6) patient BMI ≥ 30 or < 30 kg/m^2^. Summarized quantitative data for these subgroups at 1–2 weeks, 4 weeks, and 12 weeks after treatment are presented in Table 3 and Additional file 1: Tables S1 and S2. The data at 1–2 weeks after treatment showed that RF mode (RFA, WMD = − 1.76, 95% CI − 2.30 to − 1.22, *P* < 0.001), location (Asia, WMD = − 1.63, 95% CI − 2.07 to − 1.20, *P* < 0.001), site of radiofrequency (genicular nerve, WMD = − 1.64, 95% CI − 2.19 to − 1.09, *P* < 0.001), DNB (no, WMD = − 1.81, 95% CI − 2.27 to − 1.35, *P* < 0.001), sex ratio (≥ 2, WMD = − 1.59, 95% CI − 2.15 to − 1.02, *P* < 0.001), and BMI (< 30 kg/m^2^, WMD = − 1.80, 95% CI − 3.29 to − 0.31, *P* = 0.02) were potential sources of heterogeneity (all *P* < 0.05).

**Table 3:** Subgroup analysis of the WMD of pain scores between two groups at 1–2 weeks after treatment

Outcomes	No. of trials	WMD (95% CI)	Z-value	*P*	Heterogeneity	
					I^2^(%)	*P*
RF mode						
RFA	6	− 1.76 [− 2.30, − 1.22]	6.39	< 0.001	86	< 0.001
Others	4	− 1.61 [− 2.32, − 0.89]	4.39	< 0.001	40	0.17
Location						
Asia	8	− 1.63 [− 2.07, − 1.20]	7.31	< 0.001	80	< 0.001
Others	2	− 2.45 [− 3.46, − 1.43]	4.73	< 0.001	0	0.50
Site of radiofrequency						
Genicular nerve	5	− 1.64 [− 2.19, − 1.09]	5.83	< 0.001	84	< 0.001
Intra-articular	5	− 1.83 [− 2.48, − 1.17]	5.47	< 0.001	56	0.06
Diagnosed nerve block						
Yes	2	− 1.11 [− 2.40, − 0.17]	1.70	0.09	43	0.19
No	8	− 1.81 [− 2.27, − 1.35]	7.71	< 0.001	82	< 0.001
Sex ratio (female/male)						
< 2	5	− 1.51 [− 2.27, − 0.75]	3.91	< 0.001	65	0.06
≥ 2	5	− 1.59 [− 2.15, − 1.02]	5.50	< 0.001	62	0.03
BMI						
< 30	2	− 1.80 [− 3.29, − 0.31]	2.37	0.02	82	0.02
≥ 30	2	− 1.11 [− 2.40, 0.17]	1.70	0.09	43	0.19

WMD, weighted mean difference; RF, radiofrequency; RFA, radiofrequency ablation; BMI, body mass index

### Sensitivity analysis

Due to the significant between-study heterogeneity and results from subgroup analysis, we performed a sensitivity analysis to assess the stability of the pooled WMDs regarding pain score. After excluding each individual study separately, the WMDs were recalculated to identify any significant change in our results. The results of sensitivity analysis showed that the elimination of any single study was unlikely to overturn our findings (Additional file 1: Fig. S2).

## Discussion

### Main finding

Osteoarthritis is a considerable cause of disability due to the increasing prevalence of obesity and current aging of the global population [36, 37]. As a result, the development of innovative therapeutic strategies for knee OA to relieve the persistent pain and improve knee function is an important challenge. RF therapy has emerged as one of the most investigated and effective approaches in modern knee OA treatment [38]. In the present study, a meta-analysis was performed to comprehensively assess the efficacy and safety of RF in patients with knee OA. Our results suggest that the use of RF correlated with improvements in pain relief (VAS/NRS score) and knee function (WOMAC score) at four follow-up time points after treatment, but did not lead to significant improvement in the OKS. Moreover, adverse effects showed no statistically significant difference between the RF and control groups.

### Comparison with previous studies

In 2018, Hong et al. [39] performed a systematic review and meta-analysis that included 12 RCTs with 841 patients and suggested that the use of RF could decrease the pain scores (VAS) of patients at 1 week, 1 month, and 3 months after treatment, but revealed no significant improvement in knee function, which is inconsistent with the results of our meta-analysis and those of Zhang et al. [40]. Differently from our study, the negative effect on knee function in the previous systematic review by Hong et al. was evaluated by OKS, while the WOMAC score was not considered. The WOMAC score is widely applied in the evaluation of hip and knee OA for assessment of the activities of gait, daily living, general health, functional mobility, and quality of life, and has been identified as one of the highest performing outcome measures in terms of validity, reliability, interpretability, and responsiveness [41–43]. Furthermore, although the results for the effectiveness and safety of RF treatment were consistent between the current meta-analysis and previous meta-analyses by Zhang et al. [40] and Li et al. [44], the heterogeneity requires further analysis. In the present study, subgroup and sensitivity analyses were performed on RCTs to test the robustness of the pooled results and explore the potential sources of heterogeneity in our meta-analysis.

### New findings from our meta-analysis

The present meta-analysis including 1009 patients from 15 RCTs demonstrated that the use of RF treatment seemed to be effective at increasing the patient’s degree of satisfaction with the treatment effectiveness after 12-week treatment, although no statistical significance was seen at the 4-week follow-up after treatment. In addition, no SAEs were observed in any patients who received RF therapy. From our results, RF shows a seemingly excellent curative effect in patients with knee OA.

The pulse generator of RF is a simple electrode structure that generates an electromagnetic field when an electric current is passed through. RF pulse generators are able to generate frictional heat in the surrounding tissue through ionic (Na^+^, K^+^ and Cl^−^) oscillating motion repeatedly in the presence of an electromagnetic field, which in turn causes thermal destruction of the nerves and interruption of the pain impulses [45]. Meanwhile, the use of fluoroscopic or ultrasonographic guidance could ensure smooth, painless, and precise introduction of the RF cannula into the joint. This characteristic explains the immediate pain relief effect of RF therapy. However, rehabilitation training should be conducted after alleviation of the pain to increase muscle strength in the lower extremities. Patients with long-term knee joint pain may be afraid of the recurrence of pain and, thus, limit activities involving the knee joint in their daily lives [33]. Such limitation of activity may influence the measurement of patient-rated recovery, leading to a response of no significant difference in GPE scales in the short-term follow-up period. As the knee pain eases and the amount of functional activity increases, such as going up and down stairs, patients are more likely to consider their disease condition improved and report high life-satisfaction in the long-term follow-up period [33].

Our results indicated that RF treatment significantly improved knee function as assessed by the WOMAC at 4, 12, and 24 weeks after treatment, rather than the OKS. The OKS is a 12-item patient-reported outcome questionnaire regarding an individual’s level of function, activities of daily living, and how they have been affected by pain over the preceding 4 weeks. The OKS was specifically designed and developed to assess function and pain after total knee replacement (TKR) surgery or TKA [46, 47]. The WOMAC is a widely used, proprietary set of standardized questionnaires for evaluating the condition of patients with osteoarthritis of the knee and hip, including pain, stiffness, and physical functioning of the joints [48, 49]. The WOMAC is more sensitive to changes than the OKS following nonsurgical interventions for knee OA, including the effects of physical therapy, weight loss, electrotherapy corticosteroid injection, intra-articular hyaluronic acid injection, and autologous chondrocyte implantation [50–52]. In the present study, RCTs with patients who had undergone knee arthroplasty or arthroscopic surgery were excluded. Thus, it was better to apply the WOMAC to assess knee function in patients with knee OA.

Our meta-analysis suggested that pain and knee function might be alleviated by RF therapy, but the results for these curative effects were highly heterogeneous. Although subgroup analysis and sensitivity testing were performed on the pooled results, the heterogeneity was not effectively improved, which may be attributed to the following factors: (1) the inclusion criteria of Kellgren-Lawrence OA grade: the K-L grades of participants in each RCT, which ranged from 1 to 4, were relatively different. Some studies only included patients with relatively severe OA (K-L grade III-IV), while some studies included patients with relatively mild OA (K-L grade I-III). This difference in the degree of progression of knee OA will inevitably affect the difference in the efficacy of RF across the studies; (2) the intervention parameters for RF: when we re-examined the included trials, we found considerable differences in terms of RF protocols related to cycles, total operation time, and temperature, for example. Above all, differences existed even within RFA or PFA procedures [53]; (3) DNB prior to the RF procedure: innervation of the knee joint is complex and a DNB may have a role in predicting response to a RF procedure. Most of the included studies did not describe the use of DNB prior to the RF procedure. In addition, the cutoff values for the duration and amount of pain relief following DNB were not standardized within the five RCTs that reported DNB; and (4) others: the effects of RF may also be affected by the presence of local anesthetic, gender, mental health disorders, diabetes mellitus, and other conditions [54, 55].

## Limitations

Several limitations of our meta-analysis should be acknowledged. First, the data were extracted from 15 RCTs that included patients with different baseline characteristics and involved different study protocols, causing some between-study heterogeneity. Thus, further studies are needed to confirm the advantages and disadvantages of different RF protocols for patients with knee OA. Second, different follow-up time periods, multiple evaluation indices for knee function, and the lack of patient and investigator blinding among the RCTs may have caused measurement and responder biases in terms of outcomes. Third, many included studies were proof-of-concept trials with a relatively small sample size, which could decrease the statistical power. Finally, the follow-up period was relatively short in most trials, making it difficult to elucidate whether the effectiveness observed in the short- to mid-term follow-up periods could continue in the long term. Therefore, double-blind, multi-central RCTs with large sample sizes and a universally accepted RF protocol are still needed to acquire more reliable results for evaluating the efficacy and safety of RF treatment in patients with knee OA.

## Conclusion

In summary, our meta-analysis suggests that the use of RF treatment is efficacious and safe for relieving knee pain and improving knee function in patients with knee OA. However, the clinical utility of RF treatment remains poorly defined, and thus, further double-blind, multi-center RCTs of RF therapy that have large sample sizes are still needed.

## Supplementary Information

**Additional file 1: Fig. S1**. Forest plots for the assessment of OKS score between the radiofrequency group and control group. At (a) 1-2 weeks, (b) 4 weeks, and (c) 12 weeks. OKS, Oxford knee score; SD, standard deviation; CI, confidence interval. **Figure S2**. Sensitivity analysis for the assessment of pain scores at 4 weeks after treatment. SD, standard deviation; CI, confidence interval. **Table S1**. Subgroup analysis of the WMD for pain scores between the RF and control groups at 4 weeks after treatment. **Table S2**. Subgroup analysis of the WMD for pain scores between the RF and control groups at 12 weeks after treatment.

## Data Availability

All datasets generated for this research are included in this published article.

## Abbreviations

OA: Osteoarthritis
TKA: Total knee arthroplasty;
NSAIDs: Nonsteroidal anti-inflammatory drugs
HA: Hyaluronic acid
RF: Radiofrequency
RFA: Radiofrequency ablation
CRF: Cooled radiofrequency ablation
PRF: Pulsed radiofrequency ablation
RCTs: Randomized controlled trials
PRISMA: Preferred Reporting Items for Systematic review and Meta-Analysis
VAS: Visual Analogue Scale
NRS: Numerical Rating Scale
WOMAC: Western Ontario and McMaster Universities Osteoarthritis Index
OKS: Oxford Knee Score
GPE: Global Perceived Effect
WMD: Weighted mean difference
CI: Confidence interval
BMI: Body mass index
DNB: Diagnostic nerve block
SAEs: Severe adverse events
