# Supplementary material for: Efficacy and safety of radiofrequency treatment for improving knee pain and function in knee osteoarthritis: a meta-analysis of randomized controlled trials
Source: J Orthop Surg Res. 2022 Jan 15;17:21. doi: 10.1186/s13018-021-02906-4 (PMC8760716; doi:10.1186/s13018-021-02906-4)
Supplement: Supplementary file 1 — Additional file 1: Fig. S1. Forest plots for the assessment of OKS score between the radiofrequency group and control group. At (a) 1-2 weeks, (b) 4 weeks, and (c) 12 weeks. OKS, Oxford knee score; SD, standard deviation; CI, confidence interval. Figure S2. Sensitivity analysis for the assessment of pain scores at 4 weeks after treatment. SD, standard deviation; CI, confidence interval. Table S1. Subgroup analysis of the WMD for pain scores between the RF and control groups at 4 weeks after treatment. Table S2. Subgroup analysis of the WMD for pain scores between the RF and control groups at 12 weeks after treatment. [file 13018_2021_2906_MOESM1_ESM.pdf]

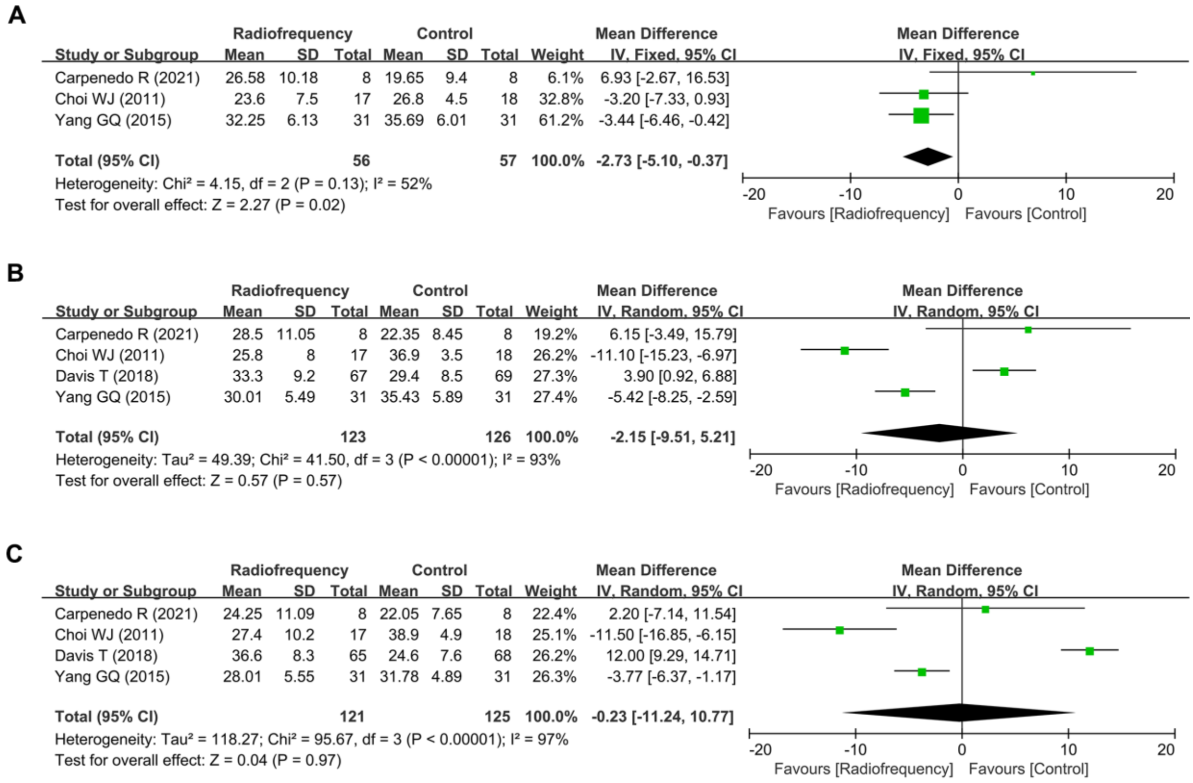

Supplementary Figure 1. Forest plots for the assessment of OKS score between the radiofrequency group and control group. At (a) 1-2 weeks, (b) 4 weeks, and (c) 12 weeks. OKS, Oxford knee score; SD, standard deviation; CI, confidence interval.

Study or Subgroup	Radiofrequency Mean	Radiofrequency SD	Radiofrequency Total	Control Mean	Control SD	Control Total	Weight	Mean Difference IV, Fixed, 95% CI	Year
Choi WJ (2011)	3.35	1.65	17	7.26	1.76	18	0.0%	-3.91 [-5.04, -2.78]	2011
Yi YS (2012)	3.41	1.67	27	4.72	1.64	30	10.8%	-1.31 [-2.17, -0.45]	2012
Rahimzadeh P (2014)	3.87	1.7	24	4.65	1.38	24	10.4%	-0.78 [-1.66, 0.10]	2014
Yang GQ (2015)	3.26	1.39	31	4.71	1.63	31	14.1%	-1.45 [-2.20, -0.70]	2015
Yuan Y (2016)	2.1	1.4	22	3.6	1.6	20	9.6%	-1.50 [-2.41, -0.59]	2016
Hu Y (2016)	2.47	0.92	45	3.94	1.37	47	35.5%	-1.47 [-1.95, -0.99]	2016
Davis T (2018)	3	2.3	67	3.9	2.2	69	14.0%	-0.90 [-1.66, -0.14]	2018
Kumaran B (2019)	2.5	1.7	15	4.4	2.3	14	3.7%	-1.90 [-3.38, -0.42]	2019
Carpenedo R (2021)	4.62	2.44	8	7	1.8	8	1.8%	-2.38 [-4.48, -0.28]	2021
**Total (95% CI)**			**239**			**243**	**100.0%**	**-1.33 [-1.62, -1.05]**	

Heterogeneity: Chi² = 4.85, df = 7 (P = 0.68); I² = 0%

Test for overall effect: Z = 9.23 (P < 0.00001)

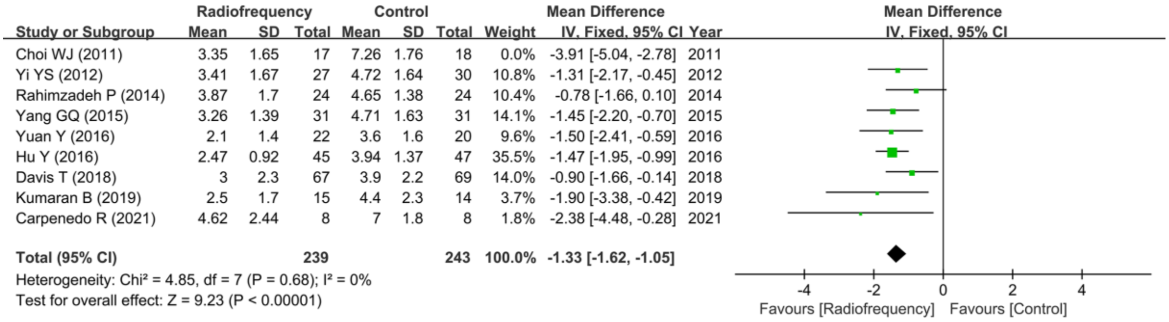

Supplementary Figure 2. Sensitivity analysis for the assessment of pain scores at 4 weeks after treatment. SD, standard deviation; CI, confidence interval.

Supplementary Table 1. Subgroup Analysis of the WMD of pain scores between two groups at 4 weeks after treatment

Outcomes	No. of trials	WMD (95% CI)	Z-value	*P*	Heterogeneity	
					I^2(%)	*P*
RF mode						
RFA	3	-2.17 [-3.61, -0.73]	2.95	0.003	87	0.005
Others	6	-1.31 [-1.64, -0.99]	7.88	<0.001	0	0.45
Location						
Asia	6	-1.66 [-2.32, -1.01]	5.00	<0.001	75	0.001
Others	3	-1.37 [-2.22, -0.52]	3.16	0.002	25	0.26
Site of radiofrequency						
Genicular nerve	4	-1.83 [-2.93, -0.72]	3.24	0.001	85	<0.001
Intra-articular	5	-1.41 [-1.77, -1.05]	7.62	<0.001	0	0.50
Diagnosed nerve block						
Yes	3	-2.37 [-4.53, -0.21]	2.15	0.03	90	<0.001
No	6	-1.38 [-1.69, -1.07]	8.78	<0.001	0	0.77
Sex ratio (female/male)						
< 2	5	-1.27 [-1.67, -0.87]	6.24	<0.001	12	0.34
≥ 2	2	-2.68 [-5.04, -0.32]	2.23	0.03	91	0.001
BMI						
< 30	3	-2.55 [-4.34, 0.77]	2.81	0.005	87	<0.001
≥ 30	2	-1.19 [-2.08, -0.30]	2.62	0.009	28	0.24

WMD, weighted mean difference; RF, radiofrequency; RFA, radiofrequency ablation; BMI, body mass index;

Supplementary Table 2. Subgroup Analysis of the WMD of pain scores between two groups at 12 weeks after treatment

Outcomes	No. of trials	WMD (95% CI)	Z-value	*P*	Heterogeneity	
					I^2(%)	*P*
RF mode						
RFA	6	-2.26 [-2.89, -1.63]	7.02	<0.001	88	<0.001
Others	5	-1.12 [-2.21, -0.04]	2.03	0.04	76	0.002
Location						
Asia	8	-1.81 [-2.49, -1.14]	5.29	<0.001	91	<0.001
Others	3	-2.08 [-2.86, -1.30]	5.25	<0.001	11	0.33
Site of radiofrequency						
Genicular nerve	6	-2.32 [-2.95, -1.68]	7.16	<0.001	88	<0.001
Intra-articular	5	-1.04 [-1.98, -0.11]	2.19	0.03	70	0.009
Diagnosed nerve block						
Yes	3	-2.65 [-3.57, -1.73]	5.65	<0.001	37	0.20
No	8	-1.58 [-2.26, -0.90]	4.52	<0.001	91	<0.001
Sex ratio (female/male)						
< 2	5	-1.61 [-2.57, -0.65]	3.28	0.001	84	<0.001
≥ 2	4	-2.38 [-3.53, -1.23]	4.04	<0.001	90	<0.001
BMI						
< 30	2	-2.79 [-4.73, -0.84]	2.81	0.005	54	0.14
≥ 30	3	-2.41 [-2.72, 2.11]	15.46	<0.001	17	0.30

WMD, weighted mean difference; RF, radiofrequency; RFA, radiofrequency ablation; BMI, body mass index;
